# The first study on seroprevalence and risk factors of *Neospora caninum* infection in pregnant local cows from Northeast Algeria

**DOI:** 10.14202/vetworld.2022.442-448

**Published:** 2022-02-25

**Authors:** Besma Abdeltif, Safia Tennah, Salima Yamina Derdour, Asma Temim, Houda Boufendi, Farida Ghalmi

**Affiliations:** 1 Research Laboratory Management of Local Animal Resources, Higher National Veterinary School, El Alia, Oued Smar, 1615, Algiers, Algeria; 2 National Center for Biotechnology Research, Ali Mendjli New Town, Constantine, Algeria

**Keywords:** Algeria, *Neospora caninum*, pregnant cattle, seroepidemiology

## Abstract

**Background and Aim:**

*Neospora caninum* is one of the most common infectious organisms worldwide that causes abortion in cattle. To the best of our knowledge, no previous studies have focused on *N. caninum* infection in the local Atlas brown cattle from Northeast Algeria. This study aimed to assess the importance of bovine neosporosis for causing abortion in Atlas brown cattle and to identify selected risk factors.

**Materials and Methods:**

A case-control study was performed on 60 control farms and 30 case farms. We collected 650 blood samples from 650 pregnant cows from 90 farms in five Algerian provinces; Jijel, Skikda, Annaba, El-Tarf, and Souk-Ahras. Sera samples were analyzed for the presence of antibodies against *N. caninum* using enzyme-linked immunosorbent assays.

**Results:**

The seroprevalence of *N. caninum* infection in the cows was 36.2% (95% confidence interval [CI]: 32.7-39.8) and in the farms was 81.1% (95% CI: 73.0-89.2). Risk factors found by multivariable logistic regression included: Presence of dogs (odds ratio [OR] 4.7, 95 CI 2.9-7.3); age ≥84 months (OR 4.9, 95 CI 2.8-8.3); Jijel region (OR 2.2, 95 CI 1.1-4.5); white (OR 2.5, 95 CI 1.4-4.4) and gray (OR 2.5, 95 CI 1.4-4.5) coat; moderate (OR 2.30, 95 CI 1.4-3.8) and bad (OR 3.1, 95 CI 1.8-5.3) hygiene; and second (OR 2.5, 95 CI 1.4-4.4); and last (OR 2.3, 95 CI 1.3-4.2) stage of pregnancy. Our case-control study showed no significant association between seropositivity of *N. caninum* and abortion at the farms level (OR 0.9, 95 CI 0.3-2.7). Similarly, there was no significant association between seropositivity of *N. caninu*m and abortion at the individual level (OR 0.8, 95 CI 0.6-1.2).

**Conclusion:**

This is the first study of *N. caninum* infection in pregnant local cows from Northeast Algeria. The prevalence rate of antibodies against *N. caninum* was high. Almost all risk factors studied for infection were significantly associated with seroprevalence. Our analysis showed no relation between *N. caninum* infection and abortion. Consequently, these local cows are resistant to abortion caused by *N. caninum*.

## Introduction

Atlas brown cattle are a local variety of cattle in Northeast Algeria near the Tunisian border [1]. They are characterized by their coat color, which is used to distinguish them. The main varieties include: Guelmoise (gray coat); Cheurfa (white coat); and Setifienne (black coat). Another local variety of cattle is the Chelfienne (red coat), which is located in Northwest Algeria. Algeria had a total of 1.8 million cattle in 2018 [2]*.*The total national cattle distribution of Atlas brown cattle in Algeria is as follows: About 59% in the northeast; 22% in the center; 14% in the northwest; and 5% in the south [1]. These cattle are characterized by low productivity due to genetic, nutritional, climatic, and health constraints.

Abortion is an economic issue for farmers. *Neospora caninum* is an apicomplexan protozoon that has been identified as a major cause of abortion in cattle worldwide, including Algeria [3,4]. Abortion is the primary clinical symptom of bovine neosporosis, with fetus death due to direct fetal or placental tissue damage between 3 and 8 months of pregnancy [5]*. N*. caninum infections have been reported worldwide [6]. Algerian studies on *N. caninum* in cattle have indicated varying prevalence levels in certain regions. Seroprevalence was 19.6% (157/799 imported, improved, and local cattle) from three provinces of North-Central Algeria [7], 12.4% (23/186 dairy cattle) from five provinces of Central-Northern Algeria [8], and 16% (23/145 cows of Prim - Holstein) from Constantine, a Northeast Algeria Province [9]. In Algiers, there was 15% (54/306 cows) seropositivity in a case-control study [4]. The seroprevalence of *N. caninum* was 2% in Turkey [10], 8.5% in Morocco [11], 11% in Italy [12], 18.9% in Egypt [13], and 21.5% in Tanzania [14]. To date, there have been no studies on the presence or absence of *N. caninum* in Atlas brown cattle in Northeast Algeria. This study aims to test the prevalence of antibodies to *N*. *caninum* and indicate risk factors associated with *N. caninum* seroprevalence in this region. A case-control study was carried out to determine the role of *N. caninum* in local cattle abortion. The aim of the case–control study is to assess the importance of *N. caninum* abortion in Algerian local cattle.

## Materials and Methods

### Ethical approval and Informed consents

No ethical approval was necessary for this study; however we obtained verbal informed consent from all farm owners involved in the study and we maintained the confidentiality of data obtained.

### Study period and areas

**Figure-1:**
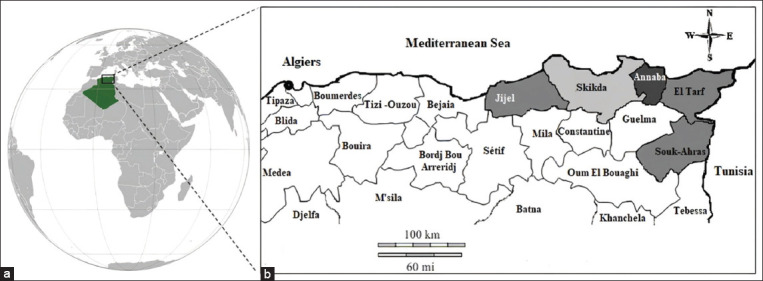
Map of the regions studied. (a) In Africa, Algeria. (b) Map of North-East Algeria; Sampled farms are indicated with gray areas [Source: modified from d-maps.com (https://dmaps.com/carte.php?num_car=4428&lang=fr)].

The study was carried out from January 2017 to September 2019. The study was conducted in five provinces: Jijel, Skikda, Annaba, El-Tarf, and Souk-Ahras. These Provinces are located in North-East of Algeria. (Latitude: 36°53’N-36°16’N, longitude: 5°46’E-9° 19’E).The geographical location of all districts selected in this study is indicated in Figure-1. The province’s climate is typically the Mediterranean. Summer is hot and dry and winter is mild and humid. This study was performed in this region because it contains more than half of the local breed cattle livestock. It is concentrated in difficult areas, particularly mountains and forests. It is living in extensive system. This system is an integral part of family farming and the national economy [15].

### Animals and sera collection

The selection criteria was pregnant Atlas brown cows. The sample size was calculated using the following formula.

N=1.96^2^ P (1−P)/D^2^.

Where N was the sample size, 1.96 was the Z value for the selected confidence level (95%), P was the individual disease prevalence, and D was the desired absolute precision. A minimum sample size of 600 animals was obtained using 50% expected individual prevalence (since there was no previous study in this area), an absolute precision of 4%, and a confidence level of 95%. However, a total of 650 animals were included in this study to increase the precision. In total, 650 pregnant Atlas brown cows were selected from 90 farms in Northeast Algeria. Five milliliters of blood were taken from the jugular vein using dry vacutainer tubes (Becton-Dickinson, USA). The sera were separated with a centrifuge and then transferred to 2 mL micro-tubes and frozen at −20°C until laboratory analysis. During farm visits, we conducted interviews with the farmers to collect data on the cattle (presence of dogs, abortion history, parity, age, coat color, and stage of pregnancy) and the farm (general hygiene, seasons, and region). Table-1 provides data collected during these visits.

**Table 1:** Risk factors associated with *Neospora caninum* with univariable analysis among pregnant local cows.

Independent variables	Categories	No. of examined cow	No. of positive cows	Seroprevalence % (95% CI)	Crude odds ratio (95% CI)	p-value
Presence of dogs	No	271	55	20.3 (15.5-28.1)	Ref.	<0.001*
	Yes	379	180	47.5 (42.5-52.5)	3.5 (2.5-5.1)	
Abortion	Yes	75	28	37.3 (26.4-48.3)	Ref.	0.82
	No	575	207	36.0 (32.8-39.9)	1.0 (0.6-1.7)	
Age classes (months)	<36	183	45	24.6 (18.3-30.8)	Ref.	<0.001*
	36-84	246	68	27.6 (22.0-33.2)	1.2 (0.8-1.8)	
	≥84	221	122	55.2 (48.6-61.8)	3.8 (2.7-5.8)	
Parity	Nulliparous	190	50	26.3 (20.0-32.6)	Ref.	<0.001*
	Primiparous	225	65	28.9 (23.0-34.8)	1.1 (0.7-1.7)	
	Multiparous	235	120	51.1 (44.7-57.5)	2.9 (1.9-4.4)	
Coat colors	Black	130	36	27.7 (20.0-35.4)	Ref	<0.001*
	Red	124	16	12.9 (07.0-18.8)	0.4 (0.2-0.7)	
	White	217	98	45.2 (38.5-51.8)	2.1 (1.3-3.4)	
	Gray	179	85	47.5 (40.2-54.8)	2.4 (1.4-3.8)	
Stage of pregnancy (months)	1-3	133	30	22.6 (15.4-29.7)	Ref.	<0.001*
	4-6	275	118	42.9 (37.1-48.8)	2.6 (1.6-4.1)	
	7-9	242	87	36.0 (29.9-42.0)	1.9 (1.2-3.1)	
Hygiene	Good	251	55	21.9 (16.8-27.0)	Ref.	<0.001*
	Moderate	215	80	37.2 (30.7-43.7)	2.1 (1.4-3.2)	
	bad	184	100	54.3 (47.1-61.5)	4.2 (2.8-6.4)	
Seasons	Autumn	140	44	31.4 (23.7-39.1)	Ref.	<0.001*
	Winter	157	49	31.2 (24.0-38.5)	1.0 (0.6-1.6)	
	Spring	200	104	52.0 (45.1-58.9)	2.4 (1.5-3.7)	
	Summer	153	38	24.8 (18.0-31.7)	0.7 (0.4-1.2)	
area	El-Tarf	61	15	24.6 (13.8-35.4)	Ref.	<0.001*
	Annaba	105	24	22.9 (14.9-30.9)	0.9 (0.4-1.9)	
	Skikda	90	19	21.1 (12.7-29.5)	0.8 (0.4-1.8)	
	Souk-Ahras	91	10	11.0 (4.6-17.4)	0.4 (0.1-0.9)	
	Jijel	303	167	55.1 (49.5-60.7)	3.8 (2.0-7.0)	

* Variables selected and used in the multivariable logistic regression model analysis (p≤0.25). Ref.=Reference, CI=Confidence interval

### Case-control study

The target population was pregnant local Atlas brown cows from Northeast Algeria. The research was carried out on each farm. A farm was considered a control if no abortion had occurred in the past 5 years. A case farm was any farm with at least one abortion in the past 5 years. In this area, two control farms of comparable size, husbandry, and management were chosen for each case farm. Data were collected from case and control farms. We used a structured face-to-face interview with farm owners to collect this data. Ninety farms were selected in this study. Thirty of them were case farms, while the other sixty were controls. In total, 650 cows were examined, including 75 cows (case cows) with recent abortion events.

### Serological analysis

Sera were tested for the presence of immunoglobulin G antibodies against *N. caninum* using an indirect multi-species enzyme-linked immunosorbent assay (ELISA) commercial test (ID Screen ® *N. caninum* Indirect Mutlti-species; ID. vet, Innovative Diagnostics, Grabels, France) at the National Center for Biotechnology Research of Constantine, Algeria. This test has a sensitivity of 99.6% and a specificity of 97.3% for the serological diagnosis of bovine neosporosis [16]. The optical density of the ELISA microplates was estimated by an automatic plate reader (PerkinElmer, Waltham, USA) at 450 nm. Seropositive animals were calculated using S/P% according to the manufacturer’s instructions. Serum with S/P% ≥50 was considered positive, and the farm was considered positive when at least one farm serum sample responded positively.

### Statistical analysis

All data were analyzed using Microsoft Office Excel 2016 and Statistical Package for the Social Sciences (SPSS) (SPSS. v.20, Chicago, IL, USA). Farm seroprevalence was determined by the ratio of positive farms to the total number of farms visited. A positive farm contained at least one seropositive cow. Cow seroprevalence was measured by the number of seropositive cows to the total number of cows examined. Confidence interval (CI) was constructed at the 95% level of confidence.

CI was calculated using the following formula:

CI=P±Pa.

P is seroprevalence obtained. Pa is absolute precision. Pa has been calculated using the following formula:


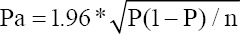


The relationship between seroprevalence and neosporosis risk factors was determined using univariable and multivariable logistic regression models [17]. The first phase was a univariable analysis of variables by a Chi-square test and crude odds ratios (OR). In the multivariable analysis, significant variables at p≤0.2 were chosen. The overall fit of the logistic regression models was tested with the Hosmer-Lemeshow test. When the OR was greater than 1 and p≤0.05, variables were considered risk factors. Multifactorial correspondence analysis (MCA) was also performed and is a graphical presentation applicable to categorical data tables in addition to logistic regression [18].

## Results

### Seroprevalence

A total of 90 farms were visited, with samples obtained from 650 pregnant local cows. Farm seroprevalence was estimated at 81.1% (95% CI: 73.0-89.2%). Cow seroprevalence was 36.2% (95% CI: 32.7-39.8%).

### Risk factors analysis

**Figure-2:**
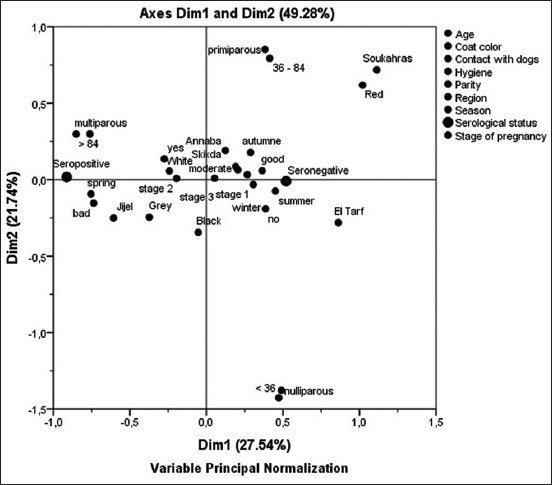
Plot of multiple correspondence analysis related to factors associated with seroprevalence of *Neospora caninum*.

As shown in Table-1, there were 11 risk factors considered significant for the risk of *N. caninum* after the univariable analysis (p<0.2): Presence of dogs; age ≥84 months; spring season; multiparous cattle; Jijel region; white and gray coat; moderate and bad hygiene; and second and last stage of pregnancy. These factors were thus selected for multivariable logistic regression analysis. The results of this analysis were: Presence of dogs (OR 4.7, 95 CI 2.9-7.3); age ≥84 months (OR 4.9, 95 CI 2.8-8.3); Jijel region (OR 2.2, 95 CI 1.1-4.5); white color (OR 2.5, 95 CI 1.4-4.4); gray color (OR 2.5, 95 CI 1.4-4.5); moderate hygiene (OR 2.30, 95 CI 1.4-3.8); bad hygiene (OR 3.1, 95 CI 1.8-5.3); second stage of pregnancy (OR 2.5, 95 CI 1.4-4.4); and last stage of pregnancy (OR 2.3, 95CI 1.3-4.2; Table-2). The model was a good fit for data (Hosmer and Lemeshow test with Chi-square=8.9; df=8; p=0.3). MCA was performed as a scatter plot to illustrate the variable groupings visually as they link to seropositivity or seronegativity (Figure-2). MCA showed that the presence of dogs (yes), white and gray coat color, second, and last trimesters of pregnancy (Stages 2 and 3), Jijel region, bad hygiene, spring season, age ≥84 months, and multiparous cattle were grouped with seropositivity. In contrast, the absence of dogs (no), autumn, winter, and summer seasons, Annaba and Skikda region, first trimester of pregnancy (Stage 1), and good and moderate hygiene were grouped with seronegativity. Other factors were not associated with the serological results: Red coat, black coat, Souk-Ahras region, El-Tarf region, age <36 months (<36), age between 36 and 84 months, primiparous cattle, and nulliparous cattle.

**Table 2:** Risk factors (logistic regression final model) associated with *Neospora caninum* seropositivity in pregnant local cows from North-Eastern Algeria.

Risk factor	B	SE	Odds ratio	Confidence interval 95%	p-value
Presence of dogs	1.5	0.2	4.7	3.0-7.3	<0.001
Age≥84 months	1.6	0.3	4.9	2.8-8.3	<0.001
Jijel area	0.8	0.4	2.2	1.1-4.5	<0.001
White color	0.9	0.3	2.5	1.4-4.4	<0.001
Grey color	0.9	0.3	2.5	1.4-4.5	<0.001
Moderate hygiene	0.8	0.2	2.3	1.4-3.8	<0.001
Bad hygiene	1.1	0.3	3.0	1.8-5.3	<0.001
Second stage of pregnancy	0.9	0.3	2.5	1.4-4.4	<0.001
Last stage of pregnancy	0.8	0.3	2.3	1.3-4.2	<0.001

B=Regression coefficient, SE=Standard error, Likelihood ratio 591.2, Chi-square goodness of fit test=8.9; df=8; P*=*0.3

### Case-control study

Forty-nine farms (81.67%) out of 60 control farms were seropositive to *N. caninum*, and 11 farms (18.3%) were seronegative. Twenty-four farms (80%) out of 30 case farms were seropositive, and six farms (20%) were seronegative. In the case and control farms, seroprevalence varied between 0% and 100%. As shown in Table-3, the link between seroprevalence and abortion was calculated by an ORs. The OR (0.9, 95% CI: 0.3-2.7) did not differ from one, indicating that there was no association of seroprevalence to *N. caninum* and the presence of abortion events on farms. Seroprevalence to *N. caninum* was the same in the control farms and case farms.

**Table 3:** Association between seroprevalence and abortion at the farms level.

Test	Case farm	Control farm
Seropositive	24	49
Seronegative	6	11
Total	30	60
Seroprevalence	80%	81.7%
Odds ratio (confidence interval 95%)	0.9 (0.3–2.7)	

Of the 650 cows examined, 575 cows had not aborted (control cows) and 75 cows had recently aborted (case cows). Two hundred and sixty-one cows (40.1%) were from case farms, and 389 (59.9%) were from control farms. As shown in Table-4, the OR (0.8, 95 CI 0.6-1.2) did not differ from one in the case-control study at the individual level. The seroprevalence to *N. caninum* was the same in the control and case cows. Thus, seropositivity to *N. caninum* was not associated with cases of abortion in cattle; therefore, exposure to *N. caninum* is not synonymous with abortion.

**Table 4:** Association between seroprevalence and abortion at the individual level.

Test	Cows from case farm	Cows from control farm
Seropositive	89	146
Seronegative	172	243
Total	261	389
Seroprevalence	34.1%	37.5%
Odds ratio (confidence interval 95%)	0.8 (0.6–1.2)	

## Discussion

Our research identified the existence of *N. caninum* for the 1^st^ time in pregnant local cows in Algeria. This research was performed using an appropriate sample (650 cows) to determine cattle and farm seroprevalence and risk factors of *N. caninum* infection in Northeast Algeria. Furthermore, a case-control study was conducted to determine the association between contact with *N. caninum* and abortion. Individual seroprevalence was 36.2% (235/650). This high seroprevalence is due to persistent contact between local cows and the parasite. This rate is higher than the seroprevalence rate previously reported in Algeria [4,7-9]. The seroprevalence in this study was higher than all seroprevalences reported in other Mediterranean and African countries: 2% in Turkey [10]; 3.4% in Nigeria [19]; 8.5% in Morocco [11]; 8.8% in Sudan [20]; 8.9% in South Africa [21]; 11% in Italy [12]; 17.9% in Senegal [22]; 18.9% in Egypt [13]; 21.5% in Tanzania [14]; 23.7% in Spain [23]; and 24.8% in pregnant cattle in Iran [24]. This variation registered might be due to the type of serological test used, the cutoff value used, region, sample size, sampling frame, farm management, contact with carnivores, breed, and climate effects, which are all known to affect results differently [25,26]. Farm seroprevalence was estimated at 81.1% (73/90), suggesting that the parasite is distributed in more than three-quarters of the studied herds. This result is higher than the 52.9% previously reported in three provinces in Northeast and Central Algeria [7]. Various studies have demonstrated substantial variance in the seroprevalence of *N. caninum* in herd cattle, including Italy 44.1% [27], Sudan 32.2% [20], and Spain 80.6% [23].

Seropositivity risk factors in this study included: presence of dogs; aged ≥84 months; originating in the region of Jijel; white color; gray color; average and poor hygiene; and the second and last stages of pregnancy. Seroprevalence increases when dogs are present; dogs are definitive hosts. *N. caninum* contaminates feeding areas of infected dogs with oocysts; thus, cattle are infected horizontally through ingestion of the infectious oocysts shed by dogs [28]. In our research, seroprevalence increased with age and was high in animals aged equal to or more than 84 months. This suggests that horizontal transmission in this population was more prevalent than vertical transmission. The previous literature has shown an association between age and seroprevalence in bovine neosporosis; nevertheless, this association is conflicting. Jensen *et al*. [29] suggested that age increases the risk of infection, but Sanderson *et al*. [30] reported that cows under 3 years old had antibody titers to *N. caninum* that were higher than cows over 6 years old.

For the 1^st^ time, the correlation between neosporosis infection and various cow colors was tested, with a significant difference between seropositivity and cow color. Gray and white-coated cows were more susceptible to infection, and the Jijel region cows are gray and white. In addition, the relationship between seropositivity and Jijel region was positive. This result needs to be interpreted carefully. Cows in the Jijel region live in an extensive system in mountains and forests, where various animals (dogs, wild canids, and cows) coexist, making horizontal transmission easier.

Hygiene also played a role, with the number of seropositive cows high on farms with poor hygienic conditions. A significant difference was found in farms with bad and moderate hygienic conditions than farms with good hygienic conditions. Improper cleaning increases feed and water contamination risk with *N. caninum* oocysts.

A significant difference in seroprevalence was also observed in gestational stage. Cows in the first trimester of pregnancy (1-3 months) had a low seroprevalence compared to cows in the second and last trimesters of pregnancy (≥4 months). This indicates that the immune system of pregnant cows is more exposed to *N. caninum* during the middle and late stages of pregnancy than in the first stage. This exposure to *N. caninum* could be due to re-infection or reactivation of the encysted parasites in tissue after the initial infection. Globally, multiple studies have suggested that cows seropositive to *N. caninum* have a higher risk for abortion than seronegative cows, including Algeria [7,24,25,31,32]. The case-control studies performed to determine the relationship between *N. caninum* antibodies and abortion in animal farms previous case-control study results have shown a significant association between seropositivity and abortion at the farm level (OR 0.9, 95 CI 0.3-2.7) and the individual level (OR 0.8, 95 CI 0.6-1.2). However, the findings of this study do not support the previous research, as the OR values varied significantly from one. The OR varied from 2.2 [33], 2.56 [4], 3.36 [34], 8 [35], 12.0 [7] to 22.1 [27] in this research. Thus, abortion episode occurrence does not link to seropositive animal presence in Atlas brown cows of Northeast Algeria.

## Conclusion

This is the first study of *N. caninum* infection in pregnant Atlas brown cows in Northeast Algeria. Seropositivity to *N. caninum* was high in these cows. Risk factors for the occurrence of antibodies to *N. caninum* included dog, age, location, color, and hygiene. Horizontal transmission of *N. caninum* is probably the most important mode of infection in this region. Our analysis showed no relation between *N. caninum* infection and abortion; thus, neosporosis should not be used as a differential diagnosis for abortion in these cattle. We recommend increasing the geographical area of study to confirm the conclusions obtained in this study. Research on the mechanism of resistance of Atlas brown cows to abortions will have to be done by genetic study. The future study should be based on identifying the gene(s) involved and the underlying mechanisms.

## Authors’ Contributions

FG, ST: Planned the study. BA: Collected samples, provided serological analyses, analyzed the data, and wrote the manuscript. SYD, AT, and HB contributed to serological analyses. FG: Supervised the study and revised the manuscript. All authors read and approved the final manuscript.
